# Physicochemical and Bioactive Stability of a Beetroot–Tarragon Microgreen Beverage During Refrigerated Storage

**DOI:** 10.3390/molecules31132247

**Published:** 2026-06-25

**Authors:** Tamara Tultabayeva, Kadyrzhan Makangali, Assem Sagandyk, Aruzhan Shoman, Damilya Konysbayeva, Zeinegul Sabitova, Kalamkas Dairova

**Affiliations:** 1 International Center, University of California, Davis, CA 95616, USA; ttultabayeva@ucdavis.edu; 2 Department of Food Production, Saken Seifullin Kazakh Agrotechnical University, Astana 010000, Kazakhstankalamkasdairova685@gmail.com (K.D.); 3 Scientific and Innovation Center Agro Tech, Astana IT University, Astana 010000, Kazakhstanzika.sabitova@gmail.com (Z.S.)

**Keywords:** green extraction, beetroot, tarragon, plant drink

## Abstract

Consumers are looking for plant-based drinks that provide natural colour and bioactive compounds. Microgreens can be used as a source of pigments and phenolics for such beverages. This study developed a beetroot–tarragon microgreen beverage using hydroalcoholic extracts obtained with a green extraction approach and examined its stability during refrigerated storage. The drink was evaluated for proximate composition, water activity, colour parameters (CIE L*a*b*), microbiological quality and antioxidant activity by the ABTS radical cation decolorization assay (ABTS) over 15 days at 4 ± 2 °C. The beverage showed low fat and energy content and water activity values close to 1.00, so microbiological safety relied on pasteurization and cold storage. *Escherichia coli* and *Staphylococcus aureus* were not detected, while total aerobic mesophilic counts reached 10^4^–10^5^ colony-forming units per gram (CFU/g), with slightly lower values in samples containing tarragon. Colour measurements indicated betalain loss and colour fading in the beetroot drink, whereas the reduction in E* was more than 80 percentage points lower in the beetroot–tarragon beverage than in the beetroot-only drink, indicating a strong protective effect of tarragon microgreens on colour stability. For the mixed beetroot–tarragon beverage, mean TEAC increased by about 37% between day 1 and day 10 of refrigerated storage. These results indicate that beetroot and tarragon microgreen extracts can be used to formulate refrigerated plant-based beverages with acceptable colour, microbiological safety and antioxidant capacity.

## 1. Introduction

Consumers’ demand for foods and beverages enriched with natural bioactive compounds has markedly increased in recent years, driven by interest in health-promoting ingredients and clean-label products [1,2,3]. Consequently, plant extracts rich in phenolic compounds and natural colorants have been widely explored as functional ingredients for the development of value-added beverages and other food products. At the same time, there is growing pressure to design extraction processes that are not only efficient but also aligned with the principles of green chemistry and sustainability [3,4,5,6].

In recent years, microgreens have emerged as a distinct class of edible seedlings obtained from vegetables and herbs harvested shortly after cotyledon expansion. Compared with their mature counterparts, many microgreens show higher concentrations of vitamins, phenolic compounds, carotenoids, betalains and other phytochemicals on a fresh weight basis, together with intense color and characteristic flavor. These properties make microgreens attractive ingredients for functional foods and beverages, especially when the goal is to deliver high levels of bioactive compounds in small serving sizes.

In this context, beetroot and tarragon microgreens combine the betalain pigments and phenolic antioxidants typical of red beet with the phenolics and volatile constituents responsible for the antioxidant and antimicrobial activities of tarragon. Using microgreen extracts instead of extracts from mature plants could therefore enhance the density of bioactive compounds and pigments in the resulting beverage, while also aligning with trends toward controlled indoor cultivation and smart, resource-efficient agriculture [1,4,6].

Green extraction of natural products aims to design processes that minimize energy input, favor safe and renewable solvents, and preserve the integrity of bioactive compounds, in line with green chemistry principles. Chemat et al. proposed six principles of green extraction, emphasizing the use of innovative technologies, process intensification, agro-solvents such as bioethanol, and energy-saving strategies as central elements of sustainable extraction processes [5]. Recent reviews emphasize that green extraction of plant polyphenols is moving from empirical solvent screening toward mechanism-driven strategies that couple suitable green media with process intensification techniques [7,8].

Plant polyphenols are among the most extensively studied bioactive compounds, due to their antioxidant, anti-inflammatory and other health-related functions, as well as their technological roles in foods. However, conventional extraction methods based on organic solvents and long processing times often face a trade-off between yield, energy consumption and polyphenol stability [9,10,11,12]. Accordingly, a wide range of green extraction strategies has been proposed to enhance polyphenol recovery while complying with green chemistry principles. Among these, hydroalcoholic ethanol–water mixtures are particularly attractive for food uses, as they provide a simple and scalable green solvent system compatible with beverage formulations [7,11,12].

Hydroalcoholic ethanol–water mixtures remain among the most widely used green solvent systems for the extraction of phenolic compounds and natural colorants, particularly in food applications. In parallel, ultrasound-assisted extraction has been recognized as an energy-saving green technology that enhances mass transfer through cavitation, allowing shorter extraction times and improved polyphenol yields. The combination of green solvent systems with ultrasound or other physical-field-based intensification has been successfully applied to plant matrices such as green tea, Hyssopus officinalis and Elaeagnus angustifolia, yielding extracts with enhanced antioxidant activity compared with conventional methods [7,10,11,12,13,14].

Beyond extraction itself, increasing attention has been paid to the incorporation of green-extracted plant polyphenols as natural colorants, antioxidants and antimicrobial agents in foods and beverages. Such applications are particularly relevant for functional drinks, where color stability, antioxidant capacity and microbial safety during storage are critical quality attributes [6].

Red beetroot (*Beta vulgaris* L.) is recognized as a rich source of betalain pigments and phenolic compounds, which exhibit strong antioxidant, anti-inflammatory, antihypertensive and potential chemopreventive effects [15,16,17]. Beetroot extracts have been proposed as natural colorants and functional ingredients in diverse food products due to their intense red color and health-promoting properties [18,19]. Recent studies also demonstrated that storage conditions markedly affect betalain stability, color attributes and antioxidant capacity in beetroot juices and concentrates, highlighting the need to control such changes during product shelf-life [14].

Tarragon (*Artemisia dracunculus* L.) contains phenolic compounds and essential oil constituents that contribute to its antioxidant and antimicrobial activities [20,21]. Tarragon extracts and essential oils have shown inhibitory effects against foodborne bacteria and have been evaluated as natural additives in products such as meat systems and bakery goods, where they can simultaneously contribute to oxidative stability and microbial control [22,23]. These properties make tarragon a promising candidate for the formulation of functional foods and beverages with extended shelf-life.

While beetroot-based beverages have been investigated with respect to color stability and changes in betalain and phenolic content during storage, and tarragon extracts have been studied as natural antioxidants and antimicrobials in meat and bakery products [20,22], there is very limited information on beverages formulated from beetroot and tarragon microgreen extracts. To the best of our knowledge, no previous study has systematically evaluated the storage stability of such a drink in terms of color parameters, water activity, antioxidant activity and antimicrobial properties under refrigerated conditions.

The combination of beetroot and tarragon extracts could thus provide a naturally colored, polyphenol-rich drink with both antioxidant and antimicrobial potential, aligning with current demand for clean-label, plant-based functional beverages. Understanding how storage affects its physicochemical and bioactive attributes is important for guiding formulation of microgreen-based beverages and further development of such products.

Therefore, the present study aimed to evaluate the physicochemical and bioactive stability of a beetroot and tarragon microgreen extract drink obtained by green hydroalcoholic extraction during refrigerated storage. We hypothesised that the inclusion of tarragon microgreen extract would contribute to microbiological quality and colour retention in a low-energy beetroot-based beverage under short-term refrigerated conditions. This work should be regarded as a preliminary feasibility study aimed at characterizing basic quality and safety changes in the beverage during refrigerated storage, rather than a full kinetic modelling investigation.

## 2. Results

### 2.1. Determination of Nutritional Profile

The proximate composition of the microgreen extracts and the model drink is summarized in Table 1.

The proximate composition of the microgreen extracts and the model drink is summarised in Table 1. All samples were characterised by negligible fat content and low energy density (≈28–29 kcal/100 g DW), with carbohydrates and proteins as the main contributors to caloric value. The beetroot–tarragon drink showed an intermediate macronutrient profile relative to the individual extracts, reflecting its mixed formulation.

### 2.2. Determination of Water Activity

**Figure 1:**
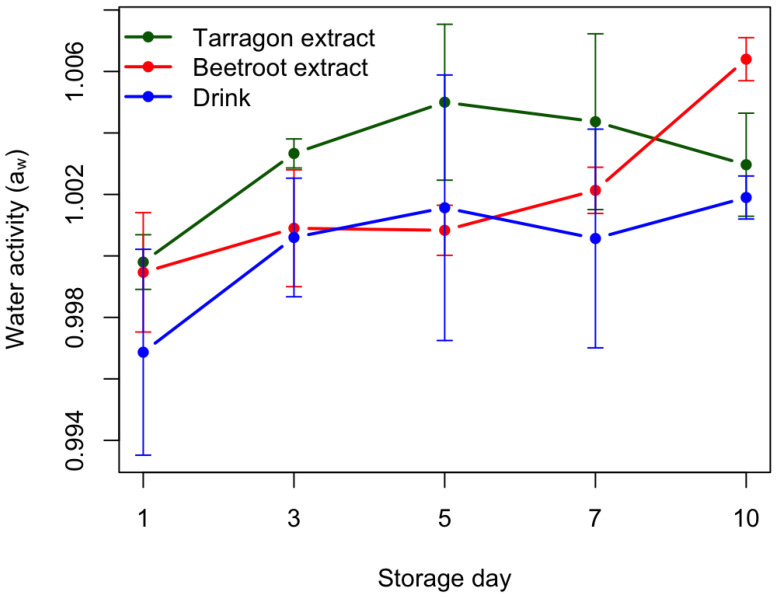
Water activity results of the samples.

During refrigerated storage, the water activity of all samples remained high and practically unchanged (Figure 1).

During refrigerated storage, the water activity of all samples remained high and effectively constant, with a_w_ values around 0.997–1.006 across the 10-day period (Figure 1). Day-to-day fluctuations did not exceed experimental standard deviations, indicating that incorporation of microgreen extracts and storage did not substantially affect water activity.

Overall, these results indicate that incorporation of microgreen extracts and subsequent 10-day refrigerated storage do not lead to any substantial changes in water activity, which is consistent with the behavior expected for high-moisture vegetable beverages and extracts.

In this study, water activity was monitored not as a hurdle for microbial growth, but to confirm that incorporation of microgreen extracts and refrigerated storage did not induce any unexpected changes in the available water of the system. As expected for high-moisture plant beverages, all samples showed a_w_ values close to 1.00 throughout storage, indicating that microbial stability could not rely on a_w_ reduction and had to be ensured instead by pasteurisation, acidity and the intrinsic antimicrobial properties of tarragon extract. Thus, the a_w_ data mainly serve to characterise the beverage matrix and to support the interpretation of microbiological results, rather than to predict shelf-life on their own.

### 2.3. Determination of Color Measurement

**Figure 2:**
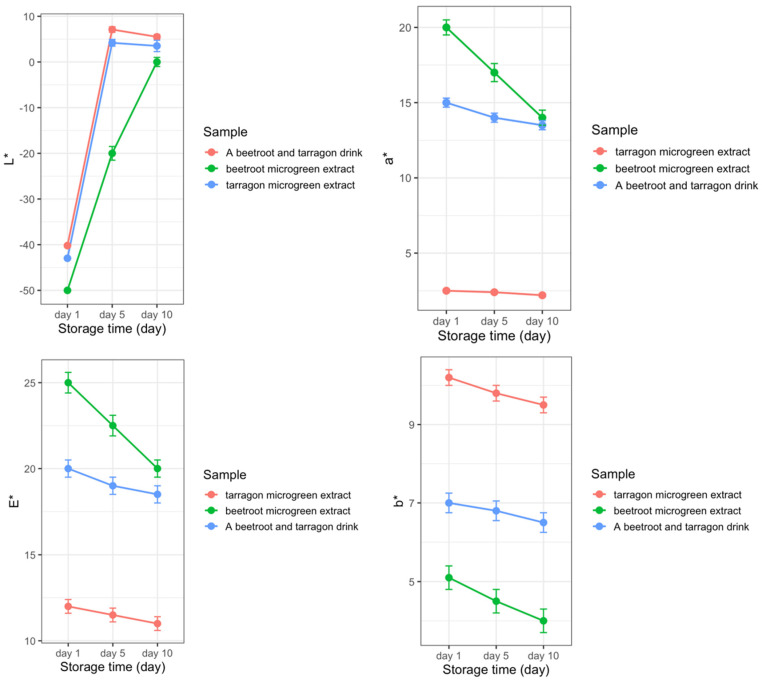
Color measurements of samples.

The color parameters of all drinks changed significantly during refrigerated storage. In the tarragon microgreen extract drink, L* and E* decreased sharply after day 5, whereas a* and b* showed only minor variations over time (Figure 2).

During refrigerated storage, all drinks exhibited noticeable changes in instrumental colour parameters (L*, a*, b*, E*; Figure 2). The tarragon microgreen extract drink mainly showed pronounced changes in L* and E*, consistent with gradual darkening and loss of vividness, while a* and b* remained relatively stable and hue was largely preserved. In contrast, the beetroot drink displayed a marked decrease in E* accompanied by shifts inof a* and b* towards zero, reflecting progressive fading of red–yellow betalain tones. The mixed beetroot–tarragon drink showed intermediate behaviour, with smaller changes in E* and colour coordinates than the beetroot drink, suggesting better visual colour retention.

### 2.4. Determination of Microbial Safety

The microbiological quality of the tarragon extract, beetroot microgreen extract and the combined beetroot–tarragon drink was monitored during storage using CompactDry plates for total aerobic mesophilic count (TC), *Staphylococcus aureus* (XSA) and coliforms/*E. coli* (CF) (Table 2).

On day 1, all samples had total aerobic counts below the detection limit and were free of *E. coli* and *Staphylococcus aureus*. During refrigerated storage, total aerobic mesophilic counts gradually increased to 10^4^–10^5^ CFU/g by day 10, while *E. coli* and *S. Aureus* remained undetectable in all cases. The final levels of total counts complied with microbiological criteria for pasteurised juice products, indicating that the extracts and beetroot–tarragon drink remained microbiologically safe throughout storage.

Despite the growth of background microflora, the absence of *Staphylococcus aureus* and *E. coli* in all samples at all time points indicates that the applied pasteurisation regime combined with refrigerated storage maintained the microbiological safety of the beverages. The combination of low pH (due to citric acid), refrigeration and the intrinsic antimicrobial properties of tarragon extract likely contributed to pathogen control, while still allowing the development of non-pathogenic background microflora.

### 2.5. Determination of Antioxidant Activity (ABTS Assay)

**Figure 3:**
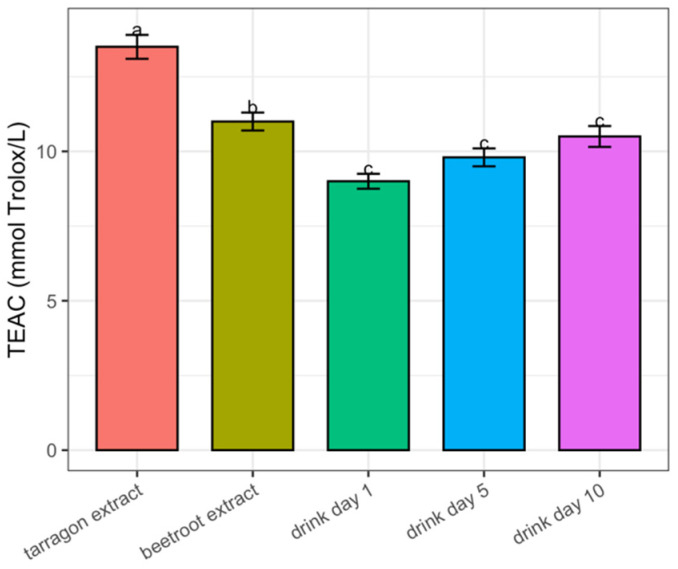
The results of ABTS assay: Colors indicate different samples: red—tarragon extract, olive—beetroot extract, green—drink at day 1, blue—drink at day 5, and magenta—drink at day 10. Bars with different letters (a–c) differ significantly according to one-way ANOVA followed by Tukey’s post-hoc test (*p* < 0.05).

The antioxidant capacity of the tarragon and beetroot extracts and the model beverages was evaluated by the ABTS assay and expressed as Trolox equivalent antioxidant capacity (TEAC, µmol TE/g dry matter). The results shown in Figure 3.

The antioxidant capacity evaluated by the ABTS assay was high for all samples, confirming that both extracts and the formulated beverages are rich in radical-scavenging compounds (Figure 3). The tarragon extract showed the highest TEAC values, followed by the beetroot extract and the beverages, consistent with its higher phenolic content reported in the literature.

## 3. Discussion

### 3.1. Nutritional Profile of the Samples

The proximate composition data indicate that both beetroot and tarragon microgreen extracts, as well as the resulting beetroot–tarragon beverage, are characterized by very low fat content and modest energy density, with proteins and carbohydrates as the main contributors to caloric value. This profile reflects the high moisture content of the microgreens and the aqueous nature of the formulation, and it is consistent with previous reports describing microgreens as low-energy foods with relatively high contents of bioactive compounds on a fresh weight basis. By providing a beverage matrix that is low in fat and energy while delivering microgreen-derived proteins and carbohydrates, the present formulation fits within the concept of plant-based drinks intended for regular consumption without substantially increasing caloric intake.

The present hydroalcoholic beetroot and tarragon microgreen extracts and the resulting beverage are consistent with the broader concept of functional foods enriched with plant-derived bioactive compounds, where health benefits are sought without a marked increase in energy density. In line with the observations of Arshad et al. and Abbasi et al., our formulations show low fat content and modest caloric value, while the potential functionality is mainly associated with microgreen-derived phytochemicals rather than macronutrients [1,2]. The choice of 40% ethanol as extraction solvent fits current definitions of green extraction, which emphasize the use of food-grade, relatively benign solvents, reduced solvent consumption and adequate selectivity toward target phytochemicals [5,7,8,9]. Chemat et al. and Lante describe ethanol–water mixtures as key examples of greener solvent systems that balance safety, polarity and extraction performance, and later reviews by Yuan et al. and Palos-Hernández et al. confirm their suitability for polyphenol recovery from plant materials in food-related applications [5,7,8,9,10,11,12,18]. Compared with more advanced green extraction approaches relying on deep eutectic solvents or subcritical water, our protocol is technologically simple but follows the same general paradigm of replacing harsher organic solvents with safer alternatives suitable for beverage formulations [7,8,9,10,11,12,18]. The use of tarragon extract is also consistent with previous reports on its antimicrobial and antioxidant activities and on its successful incorporation into food systems as a functional ingredient, which supports the feasibility of using microgreen-derived tarragon to contribute both to the functionality and the shelf-life of beverages [23,24,25,26].

### 3.2. Water Activity of the Samples

The present results on water activity are in good agreement with the literature on high-moisture plant foods and illustrate the suitability of green extraction for developing microgreen-based beverages [11,13]. Fresh vegetables, fruit and vegetable juices, and other high-moisture products typically exhibit water activity values above 0.98, often around 0.99–0.997. In this study, all microgreen extracts and the beetroot–tarragon drink showed a_w_ values close to 1.00 throughout refrigerated storage, which is fully consistent with these reported ranges and confirms that the aqueous microgreen preparations behave similarly to conventional vegetable juices [27].

Importantly, a_w_ remained effectively constant during 10 days at refrigeration temperature, with only minor fluctuations within the experimental standard deviations. This pattern aligns with previous observations that water activity in high-moisture foods changes little during cold storage unless accompanied by substantial moisture loss, solute migration, or phase transitions. From a safety standpoint, the measured a_w_ values (>0.97) indicate that all samples provide sufficient available water to support microbial growth, as also noted for most fresh and minimally processed foods. Consequently, microbiological stability of these beverages cannot rely on water activity reduction and must instead be controlled by refrigeration, acidity, and the intrinsic antimicrobial activity of microgreen phytochemicals [28].

### 3.3. Color Measurement of Samples

The present study demonstrated that the color stability of the developed drinks strongly depended on the type of plant extract used. The tarragon microgreen extract drink showed relatively moderate changes in a* and b* values during storage, indicating that the hue remained fairly stable, while the main effect of storage was a decrease in L* and E*, i.e., a gradual darkening and reduction in overall color vividness. This pattern is consistent with the phenolic and chlorophyll-type pigment profile reported for tarragon, which includes chlorogenic, caffeic and ferulic acids as well as flavonoids with antioxidant activity. These compounds can slow down drastic hue shifts but do not completely prevent lightness loss caused by oxidative processes and pigment aggregation during storage [28].

In contrast, the beetroot microgreen extract drink exhibited pronounced and statistically significant changes in all color coordinates, particularly after day 5 of storage. The increase in L* combined with the shift inof a* and b* values towards zero reflects the progressive fading of the characteristic red–yellow tone of betalain pigments and formation of less intensely colored degradation products. Similar trends have been described for beetroot juices and concentrates, where storage led to increased L* and b* values and a marked decrease in a*, corresponding to a shift from deep red–purple to yellowish-brown hues due to betanin degradation. These findings agree with studies showing that betalain stability is strongly affected by oxygen, temperature, pH and light, and that betalain degradation during storage often follows first-order kinetics [22].

The mixed beetroot–tarragon drink displayed an intermediate response between the two single-extract formulations. Although L* and E* values changed over time, the magnitude of these changes and the shifts in a* and b* were smaller than in the pure beetroot drink, suggesting partial protection of betalain pigments. This is in line with previous work demonstrating that co-presence of phenolic antioxidants can mitigate betalain degradation and improve color stability in beetroot-containing beverages and blends, such as apple–beetroot juices. In this case, the presence of tarragon extract was associated with better retention of redness and lower overall colour loss in the mixed drink compared with the beetroot-only beverage, an effect that is consistent with the known antioxidant properties of tarragon phenolic compounds, although specific interactions with betalain chromophores were not directly assessed in this study [26,29]. Quantitatively, the beetroot-only drink showed a marked loss of total colour difference (E*), with a decrease of more than 80% between day 1 and day 10, whereas in the mixed beetroot–tarragon beverage the decrease in E* over the same period was limited to about 2–3%. This indicates partial protection of colour attributes in the presence of tarragon extract; however, since phenolic content was not measured, this effect cannot be quantitatively related to phenolic levels.

In beetroot-based samples, the marked decrease in a* and E* during storage is consistent with previous reports that link betalain degradation to loss of redness and overall colour intensity in beetroot juices and concentrates [18,20,21,22,29]. Several studies have shown strong correlations between declining betanin content and decreasing a* or chroma values, supporting the use of instrumental colour parameters as indirect markers of betalain hydrolysis and oxidation [20,22,29]. In our beetroot drink, the progressive shift in of a* and b* towards zero together with the reduction in E* therefore likely reflects both cleavage of the betalain chromophore and the formation of less intensely coloured degradation products, in line with reported first-order or pseudo-first-order kinetics for betalain loss in plant beverages [19,23,30]. The mixed beetroot–tarragon drink, which showed smaller changes in a*, b* and E*, may thus be interpreted as a system where phenolic antioxidants from tarragon partially mitigate oxygen-driven betalain degradation by scavenging reactive species and stabilising the pigment structure [23,24,25,26,28].

### 3.4. Microbial Safety

In the present study, pasteurized beetroot and tarragon microgreen extracts, as well as the combined beetroot–tarragon drink, remained free of *E. coli* and *Staphylococcus aureus* during refrigerated storage, whereas the total aerobic mesophilic count increased to 10^4^–10^5^ CFU/g by the end of shelf life. This behaviour is in line with reports on functional beverages and fruit or vegetable juices, where pasteurization effectively eliminates pathogenic and indicator microorganisms but does not fully prevent the growth of residual background microflora during chilled storage. For pasteurized beetroot juices and concentrates, a similar increase in total viable counts over time has been observed without loss of microbiological safety, which closely matches the trend recorded in investigated beetroot-based samples [4,17].

The increase in total counts in products is most likely associated with the development of non-pathogenic saprophytic microorganisms (heat-resistant and psychrotrophic bacteria, yeasts and moulds) that utilise the carbohydrate-rich and phytochemical-rich matrix of beetroot juice and microgreens, as previously described for plant-based beverages and beetroot products. At the same time, the slightly lower microbial loads observed in the tarragon-containing samples are consistent with literature data demonstrating the antibacterial activity of *Artemisia dracunculus* L. essential oil and phenolic extracts in different food systems [23,26].

In our refrigerated storage trial at 4 °C, total aerobic mesophilic counts increased from undetectable levels on day 1 to 10^4^–10^5^ CFU/g by day 10, while *E. coli* and *Staphylococcus aureus* remained below the detection limit. Together with the pronounced colour fading observed in the beetroot drink, these trends suggest that, under typical consumer refrigeration in sealed packaging, a conservative practical shelf-life of about one week is advisable to ensure both microbiological acceptability and satisfactory visual quality.

Overall, these findings indicate that the combination of pasteurization, refrigeration and the addition of tarragon microgreen extract ensures the absence of target pathogens while maintaining background microflora at levels comparable to those reported for other plant-based functional beverages formulated with natural extracts.

### 3.5. Antioxidant Activity (ABTS Assay)

The antioxidant capacity of the investigated tarragon and beetroot extracts and the model beverages, expressed as Trolox equivalent antioxidant capacity (TEAC) using the ABTS assay, was relatively high, reaching several hundred µmol TE per g of dry matter. Such values are comparable to or even exceed those reported for polyphenol-rich herbal extracts and plant beverages assessed by ABTS and related methods, confirming that the selected extracts can be regarded as potent sources of natural antioxidants. The strong TEAC values are consistent with the high content of phenolic compounds previously described for spices, herbs and betalain-containing materials, which typically show a positive correlation between total phenolics and antioxidant capacity in radical scavenging assays [30,31].

A notable finding of this study is the increase in TEAC of the beverages during storage from day 1 to day 10, despite a gradual loss of the initial pink coloration and the development of a pale brown hue. Similar non-linear changes in antioxidant activity during storage have been reported for aqueous plant extracts and beverages, where an initial increase followed by a plateau or a decrease in activity was observed, depending on the solvent, temperature and light conditions. In these formulations no fermenting microorganisms were added; therefore, the observed changes are more likely related to chemical transformations and interactions between ascorbic acid and phenolic constituents rather than to microbial metabolism [32].

Several mechanisms may explain the increase in ABTS radical scavenging capacity over storage. First, ascorbic acid is a strong water-soluble antioxidant that can regenerate oxidized forms of other antioxidants, such as phenolic compounds or tocopherols, thereby enhancing the overall radical-scavenging efficiency of the mixture beyond the sum of individual components. Kinetic and model studies have demonstrated pronounced synergistic effects between vitamin C and other antioxidants, where ascorbate reduces the radical forms of co-antioxidants and maintains them in the active, reduced state, which leads to a higher and more sustained antioxidant effect in radical-scavenging systems. In mixtures of plant polyphenols and ascorbic acid, such synergy has been repeatedly shown to increase the measured antioxidant capacity in ABTS, DPPH and FRAP assays compared with single-component systems [33].

The multi-component nature of the system (tarragon extract, beetroot extract, fructose, citric acid and vitamin C) may favour complex interactions between different classes of antioxidants. Synergistic interactions among plant phenolics, organic acids and ascorbic acid have been described for a wide range of herbal and fruit matrices, where mixtures display higher antioxidant activity than predicted from individual components, particularly when assessed by electron-transfer- based assays such as ABTS [34,35]. In the experimental beverages, the progressive increase in TEAC values up to day 10 is consistent with possible interactions among ascorbic acid and other antioxidants and with the formation of additional antioxidant-active compounds during storage. Nevertheless, because individual phenolics and pigments were not quantified, the specific mechanisms underlying this behaviour cannot be confirmed and the ABTS results should be interpreted as reflecting overall antioxidant capacity rather than compound-specific effects.

Although the present work provides a detailed assessment of physicochemical stability, water activity, colour, microbiological safety and antioxidant capacity of beetroot and tarragon microgreen-based beverages, it does not include full profiling of individual bioactive compounds. In particular, betalain pigments, phenolic subclasses and volatile constituents were not quantified by chromatographic or spectrometric techniques, and the proximate composition was restricted to macronutrients rather than a complete phytochemical fingerprint. This limitation constrains the ability to directly link the observed technological behaviour and antioxidant responses to specific molecular species. Future studies should therefore address comprehensive chemical characterization of beetroot and tarragon extracts (e.g., HPLC/UPLC and GC–MS analyses of betalains, phenolic compounds and aroma components), as well as the evolution of these compounds during storage, in order to complement the present technological findings.

The storage-induced changes in TEAC can be rationalised in terms of polyphenol and ascorbic acid transformations in the beverage matrix. Similar non-linear trends in ABTS or DPPH scavenging capacity have been reported for plant beverages, where initial increases followed by stabilisation or moderate declines were attributed to the formation of new antioxidant metabolites or to gradual oxidation of phenolic constituents during chilled storage [16,17,30,31,35]. In our formulations, the relatively high TEAC values and their evolution over time are consistent with the rich phenolic background of both beetroot and tarragon extracts and with possible synergistic interactions between polyphenols and ascorbic acid, which have been shown to enhance radical-scavenging and redox-buffering capacity in model systems and complex food matrices [32,33,34]. The fact that the mixed beetroot–tarragon beetroot–tarragon drink retained or even slightly increased its TEAC despite visible colour fading suggests that loss of betalains does not necessarily translate into a proportional loss of antioxidant capacity, and that co-extracted phenolics from tarragon may sustain the overall radical-scavenging activity during refrigerated storage [16,17,30,31].

### 3.6. Limitations

This study has several limitations that should be taken into account when interpreting the results. First, the chemical characterization of beetroot and tarragon microgreen extracts was restricted to proximate composition and ABTS-based antioxidant capacity; individual phenolic compounds, betalains and other phytochemicals were not quantified chromatographically, which prevents compound-specific mechanistic conclusions about colour stability and antioxidant activity. Second, the beverage was evaluated under a single refrigerated storage condition and over a relatively short period, so the behaviour under different temperatures, light exposure or longer shelf-life scenarios remains unknown. Third, sensory properties were not assessed, and therefore no information is available on consumer acceptance of the microgreen-based drink. Future work should include detailed chromatographic profiling of phenolics and pigments, extended storage studies under varying conditions and sensory evaluation to complement the present technological findings.

Also, the beverage was evaluated only under a single refrigerated condition (4 °C) and over a short 10-day period, consistent with the intended shelf-life of a fresh, minimally processed drink without added preservatives. Consequently, the data do not allow kinetic modelling or extrapolation to longer storage times or higher temperatures.

More advanced analyses, including HPLC-based profiling of phenolic compounds and betalain pigments, are planned in the next phase of the project and will be reported in future papers.

## 4. Materials and Methods

### 4.1. Sample Preparation

**Figure 4:**
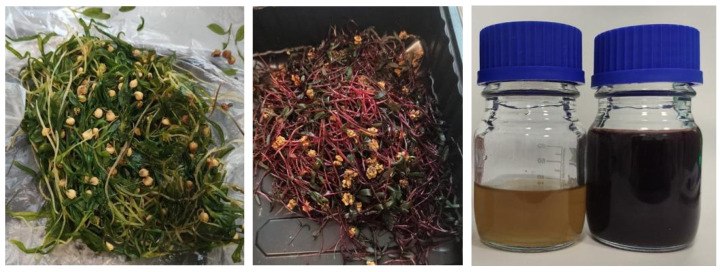
Raw materials and extracts.

A functional beverage was formulated using green hydroalcoholic extracts of beetroot and tarragon microgreens. Fresh microgreens were first harvested and washed under running tap water to remove surface impurities. The plant material was then manually ground in a mortar with a pestle to obtain a homogeneous paste. The resulting mass was mixed with 40% (*v*/*v*) ethanol at a raw material-to-solvent ratio of 1:3 (*w*/*v*) and stirred on a magnetic stirrer for 1 h at room temperature, then left in a dark place for 24 h. After extraction, the mixtures were first filtered through a paper filter (Whatman No. 1, Moscow, Russia) to remove solid plant material, and the clear filtrate was then subjected to vacuum evaporation in a rotary evaporator (Stegler, Moscow, Russia) at 30 °C until complete removal of ethanol (Figure 4). For beetroot microgreens, 45 g of fresh material yielded approximately 16.5 mL of concentrated extract, corresponding to about 7.6% total solids. For tarragon microgreens, 20 g of fresh material yielded about 16.0 mL of extract with a total solids content of 7.0%. The final beetroot–tarragon beverage contained approximately 10% total solids.

**Figure 5:**
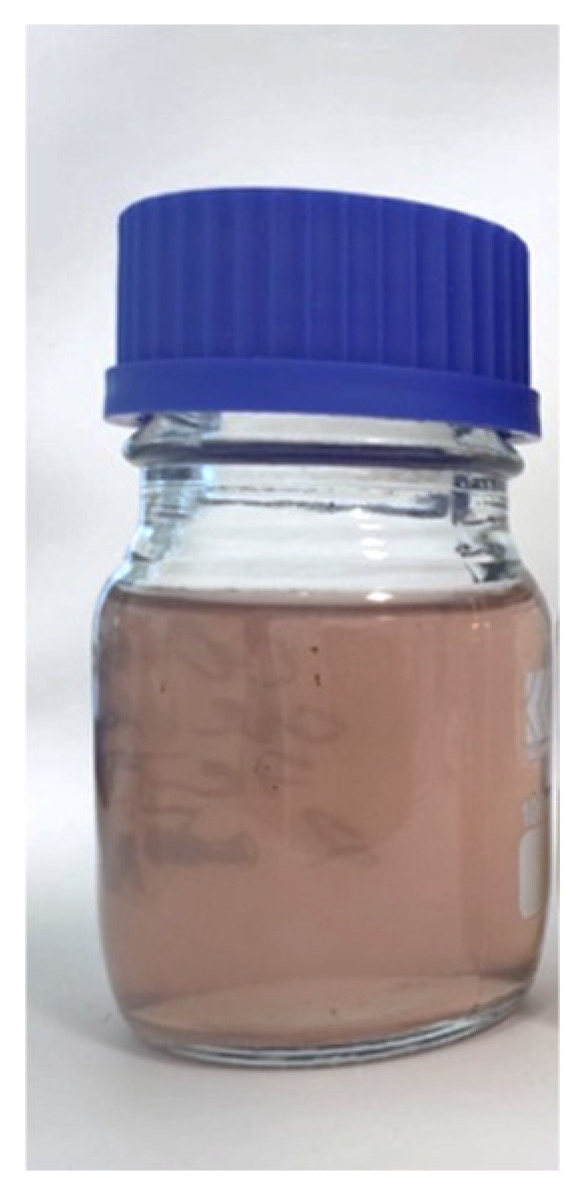
A beetroot and tarragon drink.

For beverage preparation, the extracts were combined according to the following formulation (per 100 g of product): 8 g beetroot microgreen extract, 4 g tarragon microgreen extract, 4 g fructose, 4 g citric acid, 0.2 g ascorbic acid (vitamin C), and 79.8 g water. The mixture was thoroughly homogenized and pasteurized at 65 ± 2 °C for 15–20 s, then rapidly cooled to 4 ± 2 °C and filled into sterile containers for subsequent storage and analysis (Figure 5).

### 4.2. Determination of Nutritional Profile of Samples

The proximate composition of beetroot and tarragon microgreen extracts and of the beetroot–tarragon drink, including protein, fat, ash and carbohydrates, was determined in accordance with AOAC (2016) [36] procedures. Protein content was quantified using the macro-Kjeldahl method, in which the mass fraction of nitrogen is measured and converted to protein using a factor of 6.25. Distillation and titration steps were carried out in an automatic unit (Semi-Automatic Distillation Unit, VELP, Usmate, Italy; Eco Titrator, Metrohm, Herisau, Switzerland). Fat content was measured by Soxhlet extraction (Behr behrotest R 254 S-FB, Düsseldorf, Germany) with petroleum ether as the solvent, performed in cyclic mode for 7 h. Ash content was determined by incineration in a muffle furnace (SNOL, Utena, Lithuania) at 550 ± 10 °C for 6 h. Total carbohydrates (g/100 g) were calculated by difference, using the equation:

Total carbohydrates (g/100g)=100−(m moisture+m fat+m ash+m protein)

The total energetic value was then obtained according to the formula [15]:

$$Energy\;\left(\frac{Kcal}{100}g\;DW\right)=4\times \left(m\;protein+m\;carbohydrates\right)+9\times m\;fat$$

### 4.3. Determination of Water Activity (a_w_)

Water activity (a_w_) of the beverage samples was measured using a calibrated water activity meter (AquaLab, Meter Group, Pullman, WA, USA) at 25 ± 0.5 °C. Prior to analysis, the instrument was calibrated with standard salt solutions of known a_w_ according to the manufacturer’s instructions. Approximately 5–10 g of each beverage sample were placed in the sample cup, ensuring that no bubbles or foam were present, and equilibrated in the sealed measurement chamber until a stable reading was obtained [14]. The a_w_ values were recorded in triplicate (*n* = 3) for each storage time point and expressed as mean values.

### 4.4. Color Measurement

Color parameters of the beverage were determined in the CIE L*a*b* color space using a colorimeter (Minolta CR-400, Konica Minolta, Tokyo, Japan) calibrated against a white standard tile prior to measurements. Samples were poured into clear glass cuvettes to a constant path length, ensuring absence of bubbles, and measured at room temperature. For each sample and storage time point three readings (*n* = 3) were taken at different positions, and mean L* (lightness), a* (red/green coordinate) and b* (yellow/blue coordinate) values were calculated. Chroma (C* = $\sqrt{a*2+b*2}$) and hue angle (*h*∘ = arctan(b*/a*)) were additionally computed to describe color saturation and tone [13].

### 4.5. Determination of Microbial Safety

The microbiological quality of the tarragon extract, beetroot microgreen extract and beetroot–tarragon drink was assessed using Compact Dry plates (Nissui Pharmaceutical Co., Ltd., Tokyo, Japan). Total aerobic mesophilic counts were enumerated on Compact Dry TC plates, coliforms and presumptive *Escherichia coli* on Compact Dry EC plates, and *Staphylococcus aureus* on Compact Dry X-SA plates. For each sample and storage point, 1 mL of yogurt was aseptically dispensed onto the center of the appropriate Compact Dry plate, allowing the inoculum to spread and rehydrate the dry medium in accordance with the manufacturer’s instructions. The plates were incubated at 35 ± 2 °C for 24 ± 2 h, after which characteristic colonies were counted and the results expressed as colony-forming units per gram of product (CFU/g). On EC plates, blue colonies were recorded as *E. coli*, whereas the sum of blue and red colonies was considered the total coliform count; on X-SA plates, light blue to blue colonies were interpreted as presumptive *S. aureus*. All microbiological determinations were carried out in duplicate for each batch and storage day [15].

### 4.6. Determination of Antioxidant Activity (ABTS Assay)

The antioxidant activity of the beverage was assessed using the ABTS radical cation decolorization assay, following a previously described procedure with minor modifications. Briefly, the ABTS- + working solution was prepared by reacting 7 mM ABTS with 2.45 mM potassium persulfate and allowing the mixture to stand in the dark at room temperature for 12–16 h. Before analysis, this stock solution was diluted with ethanol (or phosphate buffer) to obtain an absorbance of 0.90 ± 0.02 at 734 nm.

Beverage samples were appropriately diluted with deionized water to fall within the linear range of the assay. An aliquot (e.g., 10 µL) of diluted sample was mixed with 140 mL of ABTS- + working solution, vortexed, and incubated in the dark at room temperature for 6–10 min. The decrease in absorbance at 734 nm was then recorded using a UV–Vis spectrophotometer (Shimadzu UV-1900 spectrophotometer (Kyoto, Japan)) against a reagent blank. A Trolox calibration curve (e.g., 0–1000 µM) was used to express the results as Trolox equivalent antioxidant capacity (TEAC), in mmol Trolox equivalents per liter of beverage. All measurements were performed in triplicate (*n* = 3) and reported as mean ± standard deviation [16].

### 4.7. Statistical Analysis

All experiments were performed in triplicate, and the results are expressed as mean ± standard deviation. One-way analysis of variance (ANOVA) was used to evaluate the effect of storage time on the physicochemical and bioactive parameters of the beverage (color coordinates, water activity, antioxidant activity, microbial counts). When the ANOVA indicated significant differences (*p* < 0.05), mean separation was carried out using an appropriate post hoc test (Tukey’s HSD), and different lowercase letters (a, b, c, d) within the same row or column indicate statistically significant differences between means at the 5% significance level.

All beverages were prepared as a single production batch for each formulation. Throughout the 10-day storage experiment, bottles were randomly selected at each sampling time point and each parameter was measured in triplicate. The reported means and standard deviations therefore reflect technical replication within one batch, without additional biological replication.

## 5. Conclusions

The present study showed that hydroalcoholic extracts of beetroot and tarragon microgreens can be used to formulate a refrigerated plant-based beverage with low fat and energy content, high water activity and measurable antioxidant capacity. The drink remained free of *Escherichia coli* and *Staphylococcus aureus* during storage, and total aerobic mesophilic counts stayed within 10^4^–10^5^ CFU/g, with slightly lower values in tarragon-containing samples, indicating acceptable microbiological quality under the applied pasteurization and refrigeration conditions. Color measurements confirmed that betalain pigments from beetroot were susceptible to degradation, whereas inclusion of tarragon extract led to intermediate color changes in the mixed beverage compared with the beetroot-only formulation. All extracts and beverages exhibited high ABTS radical-scavenging activity, and antioxidant capacity was maintained or slightly increased over the storage period. Overall, these findings indicate that beetroot and tarragon microgreen extracts are suitable ingredients for developing refrigerated plant-based beverages with low energy density, controlled microbiological status and preserved antioxidant activity. The extraction and formulation protocol was intentionally kept simple (40% ethanol, conventional stirring) to align with the project goal of developing a beverage technology that can be readily transferred to commercial practice. A systematic optimisation of ethanol concentration and potential assisted extraction techniques was beyond the scope of the present work and is foreseen for future studies.

This study is limited to a feasibility-type assessment based on descriptive kinetic indicators obtained under a single storage regime. A more comprehensive, mechanism-driven kinetic analysis would require additional time–temperature combinations, extended sampling and modelling approaches, which are beyond the scope of the present work.

## Figures and Tables

**Table 1:** Proximate composition of samples, per 100 g DW.

Sample	Protein (%)	Fat (%)	Ash (%)	Carbohydrate (%)	Moisture (%)	Energy (kcal/100 g)
Tarragon microgreen extract	3.51 ± 0.12	0.00	2.34 ± 0.21	3.55 ± 0.32	90.60 ± 0.21	28.2 ± 1.4
Beetroot microgreen extract	3.30 ± 0.02	0.00	2.71 ± 0.30	3.83 ± 0.42	90.16 ± 0.30	28.5 ± 1.7
Beetroot–tarragon drink	3.41 ± 0.20	0.01	2.42 ± 0.17	3.78 ± 0.31	90.38 ± 0.17	28.9 ± 1.5

**Table 2:** Microbial counts of microgreen extracts and beetroot–tarragon drink during refrigerated storage (CFU/g).

Sample	Day of Storage	Total Aerobic Mesophilic Count	*Staphylococcus aureus*	*Escherichia coli*
Tarragon microgreen extract	1	<1 (nd)	<1 (nd)	<1 (nd)
	5	2 × 10^4^	<1 (nd)	<1 (nd)
	10	6 × 10^4^	<1 (nd)	<1 (nd)
Beetroot microgreen extract	1	<1 (nd)	<1 (nd)	<1 (nd)
	5	5 × 10^4^	<1 (nd)	<1 (nd)
	10	8 × 10^4^	<1 (nd)	<1 (nd)
Beetroot-tarragon drink	1	<1 (nd)	<1 (nd)	<1 (nd)
	5	4 × 10^4^	<1 (nd)	<1 (nd)
	10	8 × 10^4^	<1 (nd)	<1 (nd)

nd—not detected; detection limit of the method: 1 CFU/g.

## Data Availability

The original contributions presented in the study are included in the article; further inquiries can be directed to the corresponding author.
